# Japanese perspectives on transformative travel experience: humility, limitations, and dark tourism

**DOI:** 10.3389/fpsyg.2025.1592027

**Published:** 2025-06-23

**Authors:** Miho Nakajima, Takashi Oguchi

**Affiliations:** Department of Contemporary Psychology, Rikkyo University, Niiza-shi, Saitama, Japan

**Keywords:** transformative travel experience, Japan, culture, transformation scale, dark tourism

## Abstract

**Introduction:**

Although prior research has suggested that cultural background may influence the nature of transformative travel experiences, empirical studies conducted outside Western contexts remain limited. This study aims to explore how Japanese travelers experience transformative travel and to examine the cultural specificity of such experiences.

**Methods:**

A cross-sectional online survey was conducted with 864 Japanese adults, including both individuals who had experienced transformative travel and those who had not. Participants provided open-ended responses and completed the Japanese version of the Transformation Scale, which was examined for its validity and factor structure.

**Results:**

The findings indicated that Japanese travelers experienced transformative travel triggered by factors similar to those identified in Western studies, such as exposure to different values and reflective moments during travel. However, culturally distinctive outcomes also emerged, including enhanced humility and a deeper recognition of personal limitations. In addition, visits to destinations associated with dark tourism were found to promote transformative travel experiences. The factor analysis of the Japanese version of the Transformation Scale revealed a structure that differed from the original, suggesting cultural differences in how personal transformation is perceived and expressed.

**Discussion:**

These findings highlight the influence of cultural context on the nature and outcomes of transformative travel. The study contributes to a broader understanding of transformative travel experiences by revealing culturally specific elements among Japanese travelers and by validating a culturally adapted measurement scale. Future research should continue to explore cross-cultural variations in transformative travel in order to develop more inclusive theoretical frameworks.

## 1 Introduction

Transformative travel experience (TTE) refers to the process in which travel acts as a catalyst for positive changes in self-perception, attitudes, behaviors, and interactions with the world (Christie and Mason, 2003; Godovykh and Tasci, 2022; Phillips, 2019; Soulard et al., 2021). Interest in TTE has surged as the disruptions caused by COVID-19 have gradually subsided; countries have lifted their tourism and travel restrictions, and related publications have rapidly increased in recent years (Zhao and Agyeiwaah, 2023).

Several researchers have developed process theories of TTE based on the theory called “transformative learning theory” proposed by sociologist Jack Mezirow (Soulard et al., 2021; Nguyen et al., 2023; Pung et al., 2020). According to these theories, TTE typically involves three key stages: (Christie and Mason, 2003) encountering situations that challenge one’s values and worldviews, (Godovykh and Tasci, 2022) engaging in deep self-reflection, and (Phillips, 2019) arriving at new insights and obtaining transformations (Nakajima and Takashi, 2023). In everyday life, people tend to avoid situations that disrupt their comfort zones or challenge their established beliefs (Soulard et al., 2021; Hoggan, 2016). However, during travel, individuals are more likely to encounter new and unfamiliar environments that encourage a critical reassessment of their perspectives (Phillips, 2019). If travelers engage in deep self-reflection and gain meaningful insights, they may undergo transformative changes in their values and worldviews. Without such reflection, however, travelers risk reinforcing their existing biases, ultimately hindering the transformation. Thus, TTE does not automatically occur as a result of travel; rather, it depends on whether travelers actively engage in this reflective process (Soulard et al., 2021).

Zhao and Agyeiwaah (2023) reviewed the changes brought about by the TTE and classified them into four categories: psychological, behavioral, social, and spiritual. Psychological changes include shifts in values and self-perception, with TTE often helping travelers adopt more open-minded perspectives and critically examine stereotypes or narrow views (Soulard et al., 2021; Brown, 2009). Behavioral changes involve modifications to lifestyles and habits as travelers integrate new values aligned with their transformative experiences (Godovykh and Tasci, 2022; Pung et al., 2020). Social changes are frequently observed as improved communication skills and professional growth as travelers develop a global outlook and enhance their intercultural abilities (Soulard et al., 2021; Brown, 2009; Fordham, 2006). Finally, spiritual changes may include a stronger connection to nature or a heightened interest in spirituality and religious practices (Godovykh and Tasci, 2022; Soulard et al., 2021).

Transformative travel experience can occur on any type of trip as long as the trip provides an opportunity to challenge the traveler’s existing values or worldview; however, certain forms of travel are considered especially conducive to transformative experiences. For instance, leisure trips and visits to friends are often associated with TTE, whereas business travel is generally less likely to foster such experiences (Godovykh and Tasci, 2022; Tasci and Godovykh, 2021). Dark tourism is another example of travel considered particularly conducive to TTE (Zhuo et al., 2024). Dark tourism encompasses the presentation and consumption (by visitors) of real or commodified sites of death and disaster, such as trips to locations affected by nuclear accidents or war (Zhuo et al., 2024; Foley and Lennon, 1996).

## 2 Transformative travel experience and cultural backgrounds of travelers

Most research on TTE has focused on Western populations, leaving limited exploration of how cultural backgrounds shape these experiences. However, travelers’ cultural backgrounds are expected to influence the nature of TTE because of cultural differences in travel orientations and self-reflection, which is a core process in TTE (Grossmann and Kross, 2010; You et al., 2000). Indeed, some studies have highlighted notable cultural differences in TTE. For example, Teoh et al. (2024) found that Asian and Hispanic travelers in Australia developed a deeper understanding of the local culture and experienced more significant social transformation than European travelers. Similarly, Noy (2004) analyzed backpackers’ narratives and suggested that travelers’ nationalities influenced their travel experiences and identity development. These findings underscore the pivotal role of cultural background in shaping TTE.

While research on TTE among Japanese travelers is limited, it is likely that Japanese cultural factors influence the nature of their TTE, given the distinctive characteristics of Japanese travel behavior. For instance, regarding triggers for TTE, You et al. (2000) compared the travel motivations of Japanese and British travelers. They found that while British travelers prioritized gaining knowledge about destinations and engaging with locals, Japanese travelers placed less emphasis on these aspects. Additionally, Japanese travelers tend to value safety when selecting destinations (Watkins and Gnoth, 2011) and often express a strong desire to connect with nature (Watkins and Gnoth, 2011; Kankokeizai News, 2018), which also reflects their distinctive travel orientations. These cultural preferences may shape how Japanese travelers encounter and experience TTE. Building on these findings, while exposure to local culture and interactions with locals are generally recognized as common triggers of TTE (Godovykh and Tasci, 2022), such factors may function less effectively as triggers for Japanese travelers due to their lower emphasis on these aspects. In addition, stepping outside one’s comfort zone is often considered a crucial factor in TTE (Soulard et al., 2021; Nguyen et al., 2023; Pung et al., 2020). However, Japanese travelers tend to prioritize safety when selecting travel destinations (Watkins and Gnoth, 2011). As a result, their tendency to prioritize safety may reduce opportunities to encounter significantly different environments—such as geographically distant or culturally and economically dissimilar countries—that often serve as powerful catalysts for self-transformation through travel, thereby lowering the likelihood of experiencing TTE.

Conversely, studies on Japanese travel motivations suggest that Japanese travelers often embark on trips in search of a connection with nature (Watkins and Gnoth, 2011; Kankokeizai News, 2018). Given that encounters with awe-inspiring natural landscapes are closely linked to the emergence of TTE (Godovykh and Tasci, 2022; Soulard et al., 2021), it is plausible that such encounters commonly serve as triggers for TTE among Japanese travelers.

The changes brought about by TTE may also reflect unique tendencies among Japanese individuals, as suggested by previous research. For example, heightened spiritual inclination is frequently reported as an outcome of TTE (Godovykh and Tasci, 2022; Zhao and Agyeiwaah, 2023). However, Japan has a high rate of non-religiosity and a relatively low affinity for religious thought (Kobayashi, 2019; Pew Research Center, 2015). Consequently, even when Japanese travelers experience TTE, they may be less likely to develop a stronger spiritual inclination than their Western counterparts, most of whom generally have pre-existing religious beliefs (Pew Research Center, 2015).

## 3 The current study

In the present study, we investigated the cultural characteristics of Japanese travelers’ transformative travel experiences (TTE) from three perspectives based on the theoretical framework of TTE. First, we investigated the types of travel that were more or less likely to lead to TTE. Specifically, we compared the attributes of travel (e.g., travel destination, duration, primary purpose, and age at the time of travel) between those that resulted in TTE and those that did not.

Second, we examined the factors that trigger TTE and its specific outcomes by analyzing free-text data using a text-mining approach. According to the theoretical framework of TTE, individuals must first encounter situations that challenge their existing values or worldview to transform. This study aims to clarify the types of experiences that serve as triggers for Japanese travelers in particular. As mentioned earlier, encountering grand natural landscapes is expected to be a common trigger for TTE, whereas interactions with local cultures or people may be less frequent among Japanese individuals. Additionally, we analyzed free-text responses from participants who had not had a transformative travel experience, describing the most memorable travel experiences they had. By comparing these responses with those of participants who had undergone TTE, we aimed to deepen our understanding of the triggers for TTE. Furthermore, the theoretical framework suggests that TTE leads to psychological and behavioral changes in travelers. We therefore explored whether the nature of these changes reflects patterns unique to Japanese travelers. Regarding the outcomes of TTE, it is anticipated that Japanese participants will report fewer spiritual transformations than Western populations, as suggested by previous findings (Kobayashi, 2019; Pew Research Center, 2015).

Third, we examined differences in how personality perceptions, one of the outcomes of TTE, changed among Japanese travelers by validating the Transformation Scale (TS; 2). The TS is a self-report measure that evaluates the impact of TTE on personality perceptions, focusing on six traits that are commonly enhanced by TTE: Adventurousness, Compassion, Conscientiousness, Emotional Stability, Introversion, and Spirituality. Adventurousness encourages seeking new experiences and developing passion for learning. Compassion involves caring for and helping others. Conscientiousness reflects a shift from self-centeredness to disciplined behavior. Emotional Stability indicates the balance between energy and mental calmness. Introversion relates to the preference for solitude and enjoyment of time away from social interaction. Spirituality represents a strong sense of religious or spiritual inclination. The reliability and validity of this six-factor structure have been confirmed among American participants (Godovykh and Tasci, 2022). However, given that Japanese travelers may experience TTE differently from their Western counterparts, TS may exhibit a distinct factor structure for Japanese participants. In particular, cultural differences in religious and spiritual perspectives (Pew Research Center, 2015) suggest that responses to the Spirituality items may differ significantly.

## 4 Materials and methods

### 4.1 Procedure

A cross-sectional online survey was conducted in September 2022 with the cooperation of the survey firm Cross Marketing Inc. Participants were recruited from a panel of registered monitors and invited to participate in a survey titled “Study on Travel and Values.” The survey consisted of two parts: a screening survey and a main survey. Eligibility for the main survey required participants to meet all inclusion criteria outlined below.

For this study, two groups of participants were recruited: “TTE-experienced individuals” and “TTE-inexperienced individuals.” The inclusion criteria for TTE-experienced individuals were as follows: (a) 30 years of age or older, (b) having no current mental illness or ongoing psychiatric or psychological treatment, and (c) having experienced a transformative travel experience. For non-TTE-experienced individuals, the criteria included (a) and (b) above, along with (d) the absence of a TTE. The age criterion of 30 years or older was established based on research indicating that personality traits in younger populations (under 30 years) are relatively less stable (Roberts and DelVecchio, 2000), potentially increasing the influence of extraneous variables when assessing TTE outcomes. Individuals with mental illnesses or those currently undergoing treatment were excluded, as previous studies have suggested that the effects of travel may differ between individuals with and without mental health conditions (Flaherty et al., 2021). The question determined the presence or absence of a TTE, “Travel sometimes has the potential to alter one’s values and worldview. Have you ever experienced such a travel in your life?” Participants responded to this question by selecting either “Yes” or “No.”

All participants provided informed consent through an opt-out process. As part of the opt-out procedure, participants received a document that included essential information to assist them in deciding whether to participate in the survey. This document outlined the survey’s purpose, estimated time commitment to participation, voluntary involvement, and the ability to withdraw consent at any time. Additionally, it was clarified that the results would be shared at conferences and published in academic papers. However, no personal information that could identify individual participants was revealed. It was also stated that the raw data would be kept for 5 years before disposal.

Further details related to the survey methodology are presented in Supplementary Table 1 in accordance with the Checklist for Reporting Results of Internet E-surveys (Eysenbach, 2004).

### 4.2 Data cleaning

After data collection, we employed the following three procedures to detect and exclude invalid responses. First, we included an item in the questionnaire instructing participants to select a specific option (e.g., “Please choose ‘Strongly Agree’“). Responses in which options other than the instructed choice were selected were excluded from the analysis as invalid. Second, we excluded data from participants who selected the same option for all items within a single scale (e.g., consistently choosing “Strongly agree” across all items) as invalid responses. Third, for open-ended questions, we excluded data from participants who provided invalid responses, such as “I do not know.”

### 4.3 Participants

A total of 508 data points were collected from TTE-experienced individuals (male = 249, female = 259, mean age = 57.84, *SD* = 13.74), and 385 data points were collected from TTE-non-experienced individuals (male = 209, female = 176, mean age = 55.09, *SD* = 13.33). Following data cleaning procedures, the data of 482 TTE-experienced individuals were retained for analysis (male = 235, female = 247, mean age = 57.75, *SD* = 13.83). For TTE-inexperienced individuals, data from 382 participants’ data were included in the analysis (male = 207, female = 175, mean age = 55.23, *SD* = 13.29). The demographic characteristics of the participants are presented in Supplementary Table 2.

### 4.4 Ethical considerations

The research design was approved by the Research Ethics Committee of Rikkyo University (#22–26).

### 4.5 Measures

#### 4.5.1 Attributes of the travel where TTE occurred (or the most memorable travel without TTE)

For TTE-experienced participants, information was collected regarding the destination, duration, primary purpose, and age at the time of travel when the TTE occurred. TTE-non-experienced participants were asked to recall their most memorable travel experience and provide contextual details, including the destination, duration, primary purpose, and age at the time of travel. Responses indicating a travel duration of one-year or more were excluded from the analysis, as stays or visits lasting less than 1 year are generally recognized as “travel” (United Nations, 2010).

Regarding destination, the participants were first asked to specify whether the trip was domestic or international, followed by entering a specific place name. For primary purposes, participants selected one of the following seven options: vacation, visiting friends or relatives, self-development, business travel, attending school events, honeymoon, or other purposes. For the duration and age at the time of travel, the participants provided numerical responses indicating the number of days and their age during travel.

#### 4.5.2 Triggers of TTE and outcomes from TTE

This section was included in the questionnaire only for TTE-experienced participants. These participants described specific experiences from their TTE-related travels that they felt had impacted their values or worldview, according to the instruction: “What kind of experiences during your travels do you think changed your values or worldview?” They also reflected on how those experiences led to shifts in their perspectives or beliefs, as instructed: “In what specific ways do you think your values or worldview changed as a result of that trip?”

#### 4.5.3 Impressive experiences in the most memorable travel without TTE

This section was included in the questionnaire only for the TTE-inexperienced participants. These participants described the most impressive experience from their most memorable travel, according to the instruction: “What was the most impressive experience for you during the travel?”

#### 4.5.4 Transformation scale

This section was included in the questionnaire only for TTE-experienced participants. This scale measures changes in subjective personality perception resulting from the TTE (Godovykh and Tasci, 2022). After obtaining permission from the original authors, the scale was translated into Japanese. Following translation, we commissioned a specialized company (Crimson Interactive Japan Co., Ltd.) to perform back-translation to ensure consistency between the Japanese and original versions. The scale consisted of 18 items, such as “I seek more adventure than before.” Responses were rated on a 7-point Likert scale ranging from 1 (“Strongly disagree”) to 7 (“Strongly agree”).

### 4.6 Analysis

First, we compared the attributes of TTE-related travel reported by TTE-experienced participants with the most memorable travel reported by the TTE-inexperienced participants. These attributes included destination (domestic or international), duration, primary purpose, and age at the time of travel. Specifically, a chi-square test and residual analysis were used to examine the differences between destinations (domestic vs. international). Similarly, the distribution of primary purposes across the seven categories (vacation, visiting friends or relatives, self-development, business travel, school events, honeymoons, and others) was compared using the same approach. Independent Welch’s *t*-tests were conducted for the travel duration and age at the time of travel.

Subsequently, responses to three kinds of open-ended questions were analyzed using text mining, including triggers of TTE and changes brought about by TTE reported by TTE-experienced participants, as well as impressive experiences from the most memorable travels as reported by TTE-inexperienced participants. The analytical procedures were as described by Nishimura and Shimizu (Nishimura and Shimizu, 2021). Before the preliminary analysis, typographical errors were corrected and synonyms were standardized. Morphological analysis was then performed and a co-occurrence network was generated to facilitate the interpretation and categorization of the response content.

Finally, we examined the factor structure and internal reliability of the TS as well as its convergent and discriminant validity. To examine the factor structure, we first conducted confirmatory factor analysis (CFA) to evaluate the fit of the original factor structure proposed by Godovykh and Tasci (2022). The model fit was assessed primarily by focusing on CFI, GFI, and RMSEA values. For a model to be considered valid, CFI and GFI values of 0.90 or higher are generally recommended (Hair et al., 2014), while RMSEA values below 0.08 are desirable, with values under 0.10 considered acceptable (Hair et al., 2014; MacCallum et al., 1996). We also conducted exploratory factor analysis (EFA) to determine the most suitable factor structure for our data. Internal reliability was assessed using Cronbach’s alpha, with values of 0.70 or higher deemed satisfactory (Taber, 2018). Convergent and discriminant validity were evaluated based on AVE, CR, MSV, and ASV values. Convergent validity is supported if each item’s factor loading and the AVE for each subscale exceed 0.50, and CR is higher than AVE and exceeds 0.70 (Hair et al., 2014; Arifin and Yusoff, 2016). Discriminant validity was established if the AVE values exceeded greater than the MSV and ASV values (Hair et al., 2014; Almén et al., 2018).

Regarding the statistical software, text mining was performed with KH Coder, and CFA was conducted using Amos version 29. All other analyses were conducted using IBM SPSS Statistics for Windows version 29.

## 5 Results

### 5.1 Attributions of TTE occurred travel and the most memorable travel without TTE

Table 1 presents the results of the chi-square test and residual analysis for travel destinations (domestic vs. international). Whether the travel was a TTE or the most memorable travel without TTE was significantly associated with the destination (χ^2^ = 60.34, *p* < 0.01). Specifically, international travel was significantly more common in TTE travel (*p* < 0.001), whereas domestic travel was significantly more common in the most memorable travel without TTE (*p* < 0.001). The effect size was moderate (Cramér’s *V* = 0.26).

**Table 1:** Destination of transformative travel experiences occurred travel and memorable travel without transformative travel experiences.

Whether or not a transformative travel experience occurred		Domestic	International
Transformative travel experiences occurred travel	Frequency (rate in row)	153 (32%)	329 (68%)
	Adjusted residuals	−7.77***	7.77***
Memorable travel without transformative travel experiences	Frequency (rate in row)	222 (58%)	160 (42%)
	Adjusted residuals	7.77***	−7.77***

χ^2^ = 60.34***, Cramer’s *V* = 0.26. *** *p* < 0.001.

Table 2 presents the ranked lists of the most common destinations among participants who experienced TTE and the most memorable ones among those who did not. The popular travel destinations for TTE and the most memorable travel destinations without TTE were primarily the same for domestic and international trips. However, for international travel, while Thailand and Spain were among the top 10 destinations where TTE occurred, they did not appear in the top 10 for memorable travels without TTE. Similarly, for domestic travel, Hiroshima, Fukushima, and the Tohoku region were among the top 10 destinations where TTE occurred but were not included in the top 10 for memorable travels without TTE. These destinations are renowned for their connections to war and disasters; in other words, they are often associated with dark tourism (Foley and Lennon, 1996).

**Table 2:** Common travel destinations for TTE travels and the most memorable travels without TTE.

International destination							
Order	TTE occurred travels	Frequency	%	Order	Memorable travels without TTE	Frequency	%
1	USA	81	24.62	1	USA	55	34.38
2	Europe (round travel)	36	10.94	2	Europe (round travel)	14	8.75
3	Thailand	20	6.08	3	Australia	7	4.38
4	Australia	16	4.86	3	Canada	7	4.38
5	China	15	4.56	5	Korea	6	3.75
5	UK	15	4.56	5	France	6	3.75
7	Italy	14	4.25	5	Singapore	6	3.75
8	France	13	3.95	5	Italy	6	3.75
9	Korea	8	2.43	9	UK	4	2.50
9	Spain	8	2.43	10	China	3	1.88

Domestic destination							
Order	TTE occurred travels	Frequency	%	Order	Memorable travels without TTE	Frequency	%
1	Hokkaido	23	15.03	1	Hokkaido	36	9.42
2	Okinawa	17	11.11	2	Okinawa	26	6.8
3	Kyoto	8	5.23	3	Kyoto	20	5.23
3	Tokyo	8	5.23	4	Tokyo	16	4.29
3	Kyushu (round trip)	8	5.23	5	Nagano	9	2.36
6	Hiroshima	7	4.58	6	Shizuoka	8	2.1
7	Tohoku (round travel)	6	3.92	7	Mie	7	1.83
7	Shizuoka	6	3.92	8	Kyushu (round travel)	6	1.57
9	Fukushima	5	3.27	9	Aichi	5	1.31
10	Nagano	4	2.61	9	Fukuoka	5	1.31

Table 3 presents the results of the chi-square test and the residual analysis for the primary purpose of travel. Whether the travel was a TTE or the most memorable travel without TTE was significantly associated with the primary purpose (χ^2^ = 43.23, *p* < 0.01). Self-development (*p* < 0.01) and business travel (*p* < 0.05) were significantly more common in TTE travel, whereas honeymoons were significantly more common in the most memorable travel without TTE (*p* < 0.001). The effect sizes ranged from small to moderate (Cramér’s *V* = 0.22).

**Table 3:** The primary purpose of TTE occurred travel and memorable travel without TTE.

Whether or not a transformative travel experience occurred		Vacation	Visiting friends or relatives	Self-development	Business travel	School event	Honeymoon	Other
TTE occurred travel	Frequency (rate in row)	251 (52.07%)	35 (7.26%)	38 (7.88%)	34 (7.05%)	17 (3.53%)	25 (5.19%)	82 (17.01%)
	Adjusted residuals	0.22	0.57	3.19**	2.16*	−1.73	−5.26***	1.26
Memorable travel without TTE	Frequency (rate in row)	196 (51.31%)	24 (6.28%)	11 (2.88%)	14 (3.66%)	23 (6.02%)	61 (15.97%)	53 (13.87%)
	Adjusted residuals	−0.22	−0.57	−3.19**	−2.16*	1.73	5.26***	−1.26

χ^2^ = 43.23***, Cramer’s *V* = 0.22, ***p < 0.01, **p < 0.01, *p < 0.05.

In terms of travel duration, TTE travels (*M* = 13.10, *SD* = 26.89) were significantly longer than the travels without TTE (*M* = 8.97, *SD* = 32.56; *t* = 2.00, *df* = 734.17, *p* < 0.05). Regarding age at the time of travel, participants who experienced TTE travel (*M* = 34.96, *SD* = 16.04) were significantly older than those who experienced their most memorable travel without TTE (*M* = 30.10, *SD* = 14.72; *t* = 4.63, *df* = 843.69, *p* < 0.001).

### 5.2 Contents of descriptions related to TTE occurred travel and the most memorable travel without TTE

#### 5.2.1 Triggers of TTE

**Figure 1:**
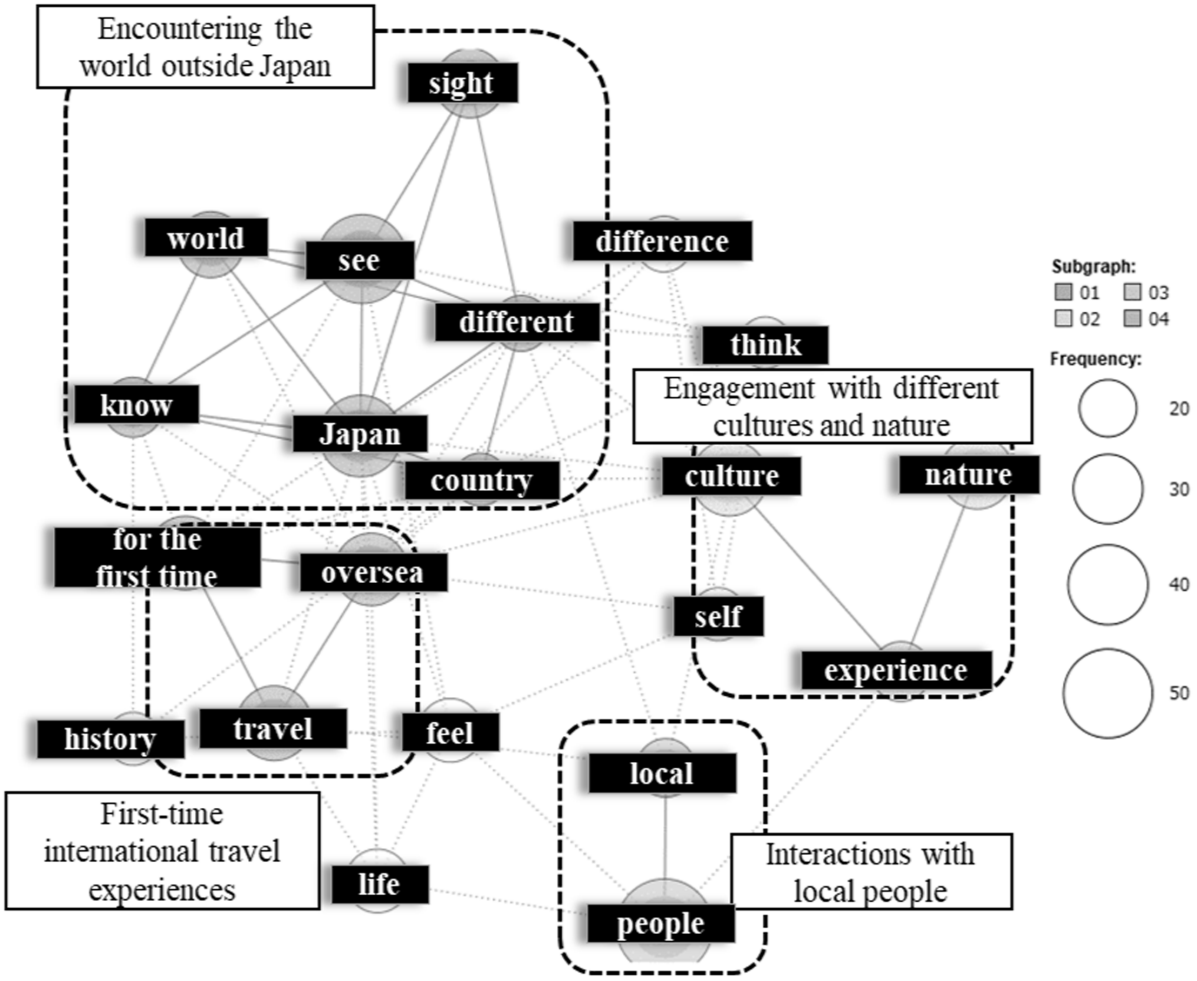
The co-occurrence network of “triggers of transformative travel experiences.” This analysis was previously presented at the 38th Annual Conference of the Japan Institute of Tourism Research (Nakajima and Takashi, 2023).

First, we analyzed the open-ended responses regarding the triggers for TTE. The top 15 most frequently extracted words are shown on the left side of Supplementary Table 3, and a co-occurrence network map is presented in Figure 1. To reduce visual clutter and facilitate the interpretation of connections among the extracted words, the co-occurrence network was limited to words that appeared 20 or more times. This analysis revealed clusters of related words. In the upper left, a cluster containing “Japan,” “see,” “know,” “world,” “country,” “different,” and “scenery” was identified. An examination of the Key Word in Context (KWIC) concordance showed that these words were used in contexts such as “seeing the scenery of countries other than Japan” and “learning about a world different from Japan.” Thus, this cluster was interpreted as reflecting “encountering the world outside Japan.” In the lower left, a grouping of “internationals,” “first time,” and “travel” was found, consistently used in the context of “first-time internationals travel experiences.” In the lower right, a cluster of “people” and “local” was observed, which appeared in contexts like “learning from local people” and “understanding the feelings of locals,” suggesting “interactions with local people.” Finally, in the upper right, a grouping of “culture,” “nature,” and “encounter” was identified, used in contexts such as “directly experiencing a foreign culture” and “being in touch with magnificent nature.” This cluster was therefore interpreted as representing “engagement with different cultures and nature.”

#### 5.2.2 Outcomes from TTE

**Figure 2:**
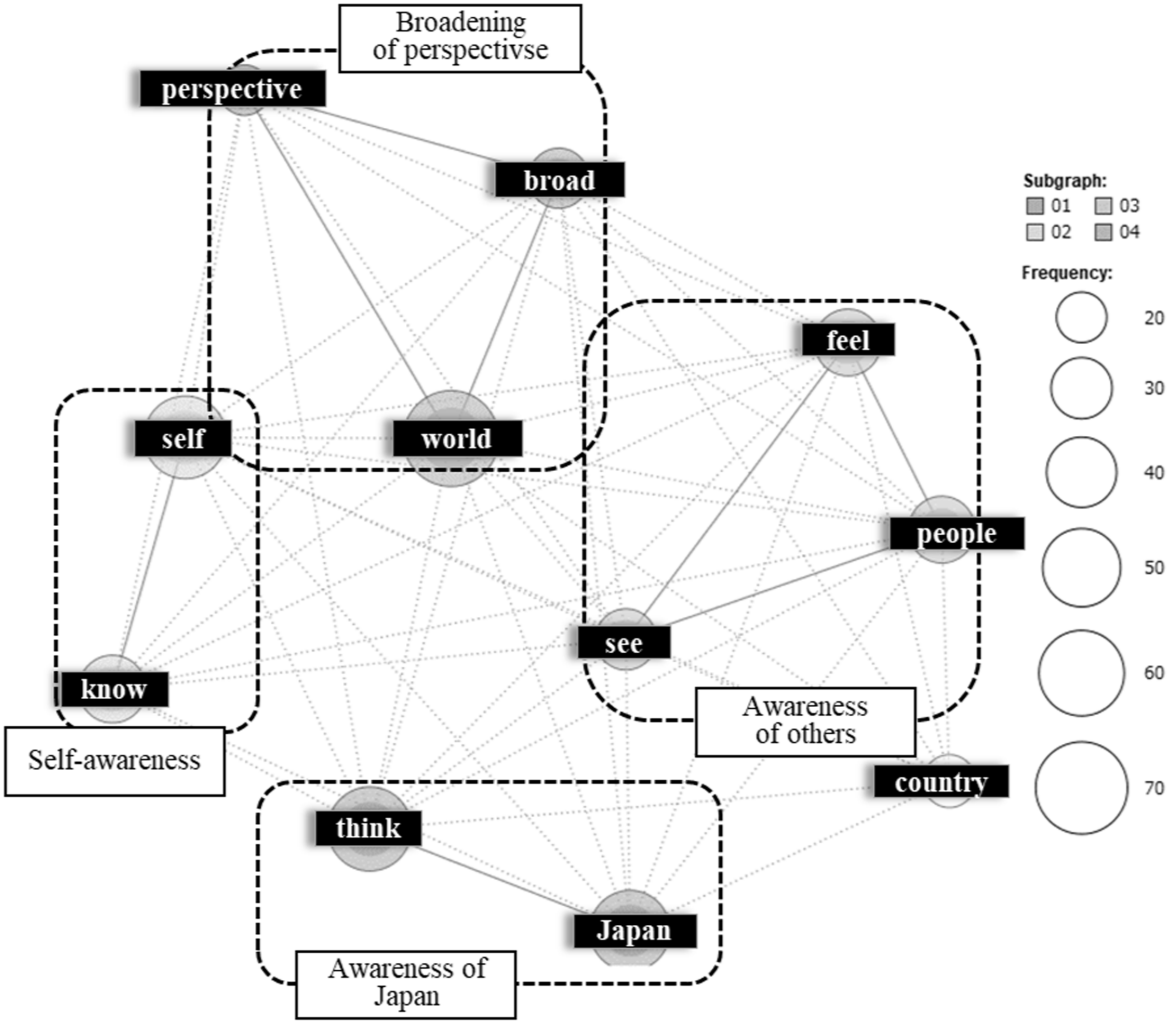
The co-occurrence network of “outcome from transformative travel experiences.” This analysis was previously presented at the 38th Annual Conference of the Japan Institute of Tourism Research (Nakajima and Takashi, 2023).

The 15 most frequent extracted terms are shown on the middle part of Supplementary Table 3. A co-occurrence network diagram is shown in Figure 2. In the upper left area, a cluster of terms such as “world,” “broad,” and “perspective” was identified. These terms were used in contexts like “I realized how vast the world is” and “My perspective broadened beyond its previously narrow scope.” Thus, this cluster was interpreted as indicating a “broadening of perspectives.” In the center left area, a cluster of terms like “self” and “see” was identified, often appearing in contexts such as “I realized how fortunate I am” and “I became aware of my own immaturity.” This statement was interpreted as representing “self-awareness.” In the center right area, terms like “feel,” “see,” and “people” formed a cluster, frequently used in contexts such as “I felt people’s warmth and sincerity” and “Seeing others made me feel that I, too, need to show kindness.” This saying was interpreted as expressing “awareness of others.” Finally, in the lower area, a cluster of terms like “Japan” and “think” appeared, used in contexts like “I thought I should learn more about Japan” and “I felt proud to be Japanese.” This cluster was interpreted as representing “awareness of Japan.”

#### 5.2.3 Impressive experiences in the most memorable travel without TTE

**Figure 3:**
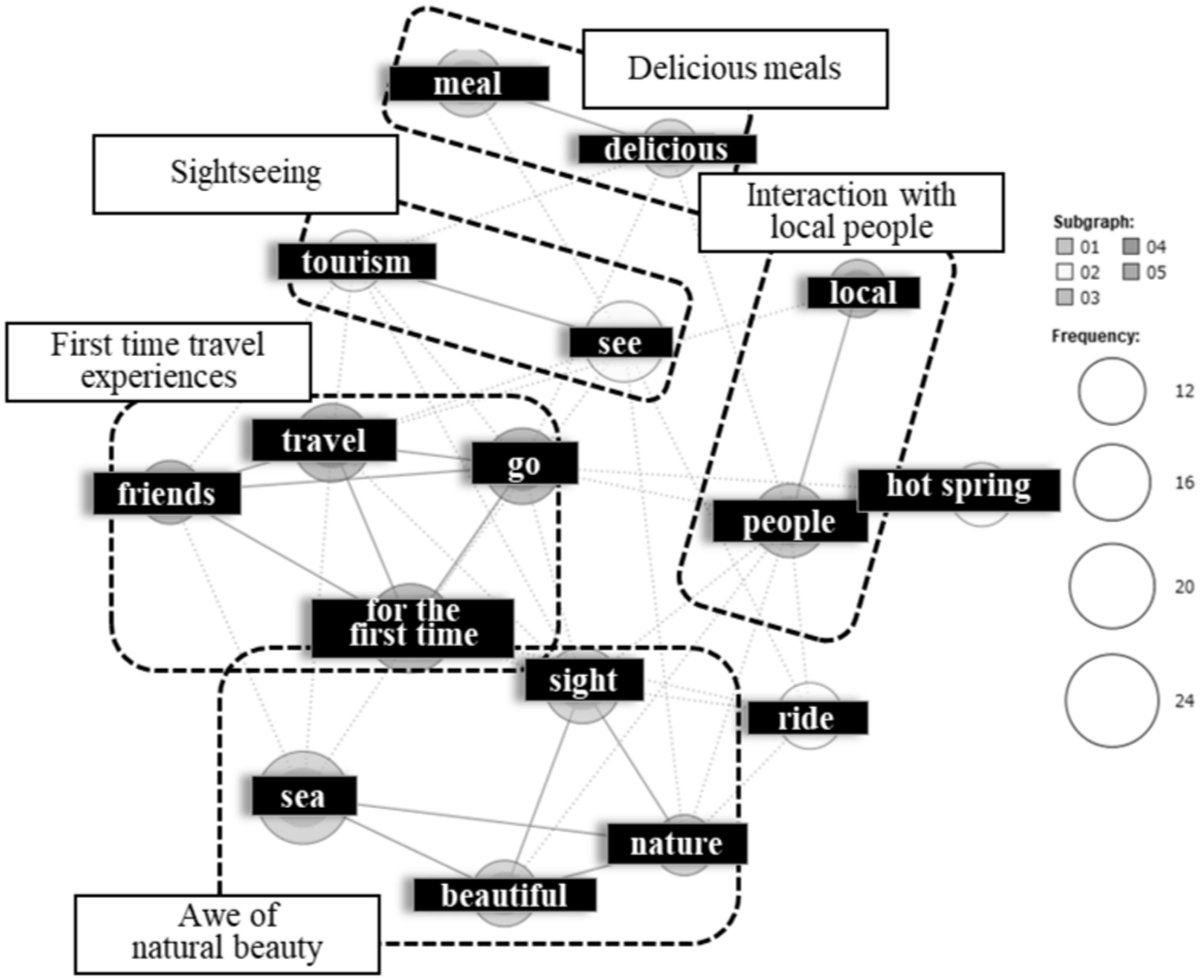
The co-occurrence network of “Impressive experiences in the most memorable travel without transformative travel experiences.”

The 15 most frequently extracted terms are shown on the right side of Supplementary Table 3, and the co-occurrence network diagram is presented in Figure 3. In the upper area, a cluster including “meal” and “delicious” was identified. As these terms consistently appeared in the context of “the meal was delicious,” this cluster was labeled “Delicious meals.” Below this, a cluster of terms “tourism” and “see” was identified, used consistently in the context of “sightseeing” and thus labeled as such. Further down, a cluster formed by “for the first time,” “go,” “travel,” and “friends” was identified, appearing in contexts such as “I traveled abroad for the first time” and “I took a travel with friends for the first time.” This cluster was labeled “First-time travel experiences.” In the right area, a cluster of “local” and “people” was identified, used in contexts like “I talked with locals” and “I enjoyed interacting with local people,” so it was labeled “Interaction with local people.” Finally, in the lower area, a cluster of “sight,” “nature,” “beautiful,” and “sea” was identified, used in contexts such as “the sea was beautiful” and “majestic nature,” leading to the label “Awe of natural beauty.”

### 5.3 Transformation scale

#### 5.3.1 Factor structure

Confirmatory factor analysis was conducted to examine the validity of the original six-factor model. The results indicated that the fit indices of the original six-factor model fell outside acceptable ranges (χ^2^ = 736.57, *df* = 120, GFI = 0.856, AGFI = 0.79, CFI = 0.864, RMSEA = 0.103).

Next, EFA was conducted to investigate the optimal factor structure of TS for the Japanese participants in this study. Prior to factor analysis, we examined whether ceiling and floor effects were present for each item. The results showed a floor effect for item Q16, “Compared to before, now I practice religion more.” However, the mean minus the standard deviation for Q16 was 0.95, which was only slightly below the minimum response value of 1; therefore, the item was retained at this stage.

In the EFA, we first examined the number of factors using the Guttman criterion and conducted a parallel analysis based on diagonal squared multiple correlations. Both criteria suggested a five-factor structure for this scale. Therefore, we conducted an EFA with maximum likelihood estimation and promax rotation, fixing the number of factors at five. After the initial analysis, items Q15, Q16, and Q18 had factor loadings below the threshold of 0.50, and Q17 had a communality of 1; therefore, these four items were removed and the analysis was repeated. Table 4 presents the results of the analysis.

**Table 4:** The factor structure of the Transformation Scale among the Japanese participants in this study.

Item		Mean	*SD*	Factor loadings
Adventurousness				
1	I seek more adventure than before.	3.97	1.55	0.65
2	I seek to visit new places more than before.	4.96	1.35	0.82
3	I seek more changes than before.	4.41	1.25	0.84
Compassion				
4	Compared to before, now I feel others’ emotions more.	4.24	1.21	0.71
5	I inquire about others’ well-being more than before.	4.00	1.27	0.74
6	I do things for others more than before.	4.24	1.22	0.80
Conscientiousness				
7	Compared to before, now I believe more that laws should be strictly enforced.	4.50	1.26	0.88
8	I try to follow the rules more than before.	4.57	1.25	0.95
9	I like order more than before.	4.37	1.28	0.85
Emotional stability				
10	I am more relaxed than before.	4.58	1.24	0.67
11	I am less easily frustrated than before.	4.15	1.25	0.87
12	I feel less frequently blue than before.	4.17	1.23	0.87
Introversion				
13	I seek quiet more than before.	4.28	1.28	0.81
14	I enjoy silence more than before.	4.01	1.27	0.84
Deleted items				
15	I enjoy my privacy more than before.	4.71	1.15	
16	Compared to before, now I practice religion more.	2.43	1.49	
17	I am a more spiritual person than before.	2.94	1.56	
18	I keep my faith even during hard times more than before.	4.11	1.26	

The items corresponding to Adventurousness, Compassion, Conscientiousness, and Emotional Stability in the original scale retained their original factor structures. For Introspection, one item (Q15) was removed from the original introspection items, leaving two items to represent this factor. Three items that constituted spirituality on the original scale (Q16, Q17, and Q18) were removed.

#### 5.3.2 Internal consistency

Cronbach’s *α* coefficients were calculated for each subscale, with all subscales demonstrating values of 0.70 or higher (Adventurousness α = 0.80; Compassion α = 0.80; Conscientiousness α = 0.92; Emotional Stability α = 0.84; Introspection α = 0.81). These results confirmed that each subscale had adequate internal reliability.

#### 5.3.3 Convergent and discriminant validity

To assess convergent and discriminant validity, the AVE, CR, MSV, and ASV values were calculated, and the results are presented in Table 5. For all the subscales, these values met the criteria for establishing convergent and discriminant validity.

**Table 5:** Values of AVE, CR, MSV, ASV in each factor.

Factor	AVE	CR	MSV	ASV
Adventurousness	0.60	0.81	0.15	0.06
Compassion	0.56	0.80	0.43	0.26
Conscientiousness	0.80	0.92	0.43	0.24
Emotional Stability	0.66	0.85	0.27	0.19
Introversion	0.68	0.81	0.26	0.17

AVE, average variance extracted; CR, composite reliability; MSV, maximum shared variance; ASV, average shared variance.

## 6 Discussion

This study examines how Japanese travelers tend to experience TTE. The findings revealed unique tendencies among Japanese travelers, and their experiences aligned with those reported in previous Western research.

### 6.1 Types of travels conducive to transformative travel experiences

This study suggests that TTE is more likely to occur during long-term international travel in Japanese individuals. This finding aligns with previous research on Western samples (Godovykh and Tasci, 2022). This result might reflect the limited novelty or cultural differences that domestic destinations offer compared to international travel.

With regard to the association between primary travel purposes and TTE, travel for self-development and business purposes was more likely to lead to TTE, whereas honeymoons were less likely to do so. The findings regarding self-development align with previous research indicating that travel focused on education or self-discovery fosters TTE (Pearce and Foster, 2007) and supports the idea that motivation for personal growth contributes to TTE (Nakajima and Takashi, 2023).

However, the results concerning business travel contradict findings from Western studies, suggesting that business travel is a type of travel that is unstable for experiencing TTE (Godovykh and Tasci, 2022). This discrepancy may reflect cultural differences in attitudes toward work. In Western contexts, greater emphasis is generally placed on maintaining work-life balance (Haar et al., 2014). Conversely, traditional Japanese values, such as lifetime employment, have historically encouraged a strong sense of loyalty to one’s organization, making work a central part of life in Japan, although such values are gradually evolving (Kito, 2014). Moreover, research on well-being has shown that dedication to work is closely linked to happiness among Japanese adults (Takagi, 2022). Given that many individuals in Japan find a sense of purpose in their work, it is possible that their strong commitment to business trips makes them more likely to experience a transformative travel experience. Future studies using robust research designs are needed to empirically investigate this potential cultural difference.

Transformative travel experience were found to be less likely to occur during honeymoons, which was not reported in previous studies. The reason for this outcome remains unclear. However, one possible explanation is that individuals on their honeymoons may primarily focus on their new spouse, diverting attention away from self-reflection or transformative experiences. Achieving TTE requires encountering factors that prompt introspection (Nakajima and Takashi, 2023). However, during a honeymoon, travelers’ attention may be predominantly directed toward their partner, which could reduce their engagement with new environments or experiences encountered during the trip.

Although vacation travel has been suggested to be conducive to TTE (Godovykh and Tasci, 2022), this tendency was not revealed in the present study. It is assumed that the methodological differences between the studies might be reflected in this discrepancy. Specifically, this study statistically compared travels that resulted in TTE with those that did not, whereas previous studies simply aggregated data from travels where TTE occurred (Godovykh and Tasci, 2022). Indeed, vacation was the most common purpose of travels with TTE, even in the present study, when the data were simply aggregated. However, as this study suggested, vacations were also the most common purpose of travel where TTE did not occur. This implies that vacations may not be advantageous for achieving a transformative travel experience.

The results also suggest that TTE-induced travel occurs at older ages than travel without TTE. This finding seems to contradict theories suggesting that flexibility in identity and values declines with age (Roberts and DelVecchio, 2000). Although the underlying reason for this is unknown, it may be related to an individual’s economic trajectory. As noted earlier, TTE tend to occur during longer international travel, which is more expensive. In Japan, older individuals generally have higher incomes owing to seniority-based wage systems (Ministry of Health, Labor and Welfare, 2022). Consequently, they may be more financially able to afford longer international trips, which tends to induce TTE. On the other hand, younger individuals in Japan may face financial constraints, making such travel less accessible and possibly limiting their opportunities for TTE.

### 6.2 Triggers of transformative travel experiences

The results identified four factors that triggered TTE among participants: engagement with diverse cultures and natural environments, interactions with local people, exposure to places outside Japan, and first-time international travel experiences. These findings align with those of previous studies (Godovykh and Tasci, 2022; Soulard et al., 2021), indicating that Japanese travelers, like Western travelers, experience TTE through similar triggers.

Interestingly, despite prior research suggesting that Japanese travelers generally prefer less interaction with local cultures and people (You et al., 2000), our results suggest otherwise. Participants frequently used terms such as “people” and “culture” in their responses (Supplementary Table 3), highlighting the significance of these interactions as potential triggers for TTE. This finding challenges the initial hypothesis that such experiences may have less of an impact on Japanese travelers. Instead, it suggests that, under certain conditions, Japanese travelers may place greater value on cultural and interpersonal engagement than previously thought.

### 6.3 Outcomes from transformative travel experiences

The analysis identified four primary outcomes of TTE: broadening perspectives, self-awareness, awareness of Japan, and awareness of others. These findings largely align with the existing research conducted in Western contexts. First, regarding the “broadening of perspectives” and “awareness of Japan,” the participants articulated how their experiences during the TTE expanded their worldview and prompted critical reflections on Japanese culture. Prior Western research has similarly suggested that engaging deeply with foreign cultures through TTE often facilitates the reassessment of one’s cultural identity and values (Soulard et al., 2021). Furthermore, concerning “awareness of others,” participants reported a newfound appreciation for the kindness of others and expressed a desire to exhibit greater kindness themselves. This outcome corresponds closely with the findings of Western studies (Godovykh and Tasci, 2022).

In contrast, the theme of “self-awareness” revealed a notable divergence from Western findings. Many participants described gaining a heightened awareness of their own immaturity and privileges, which often cultivated a more humble perspective. While Western studies have also identified a connection between TTE and increased humility, they have frequently reported a concurrent enhancement in self-confidence (Soulard et al., 2021). This apparent contrast suggests that the nature of humility fostered by TTE differs significantly between Japanese and Western travelers. One plausible explanation for the distinctive form of humility observed among the Japanese participants is the pervasive influence of Buddhist philosophy on Japanese cultural and cognitive frameworks. Although most Japanese individuals do not adhere to specific religious beliefs (e-Stat, 2023), Buddhist principles are deeply embedded in their daily practices and societal norms. For instance, Buddhist rituals are predominant in Japanese funerals, reflecting the integration of Buddhist philosophy with cultural traditions. One core tenet of Buddhism emphasizes the interconnectedness of all beings, encouraging individuals to recognize their reliance on the surrounding world for their existence (Tada, 2015). This philosophical perspective aligns with humility, characterized by an acknowledgment of one’s limitations, which emerged as a salient outcome of the TTE among Japanese participants. It is conceivable that this form of humility, marked by an awareness of one’s own immaturity, is unconsciously regarded as a virtue within the Japanese cultural context and is brought to conscious awareness through the reflective processes inherent in TTE.

From this vantage point, the enhancement of humility, specifically the recognition of one’s immaturity, can be interpreted as a spiritual transformation arising from TTE. Interpreting the above, it may not be unusual for Japanese travelers, who generally have less religious inclination, to experience spiritual changes through TTE. However, in this study, no open-ended responses explicitly used terms such as “spiritual” or “religious.” Therefore, it is predicted that even if Japanese travelers experience substantial spiritual changes through TTE, they may not recognize these changes as “spiritual.” This tendency suggests that while the transformative effects of TTE may be significant, their interpretation within the Japanese cultural context does not necessarily align with conventional Western conceptions of spirituality.

### 6.4 Transformative travel destinations

Many Japanese travelers prioritize safety (Watkins and Gnoth, 2011), leading to the assumption that they may be less likely to experience TTE in culturally distinct environments. In line with this expectation, many participants reported experiencing TTE in culturally familiar locations, such as developed countries or nearby Asian regions (Table 2). However, in our data, even when traveling to relatively culturally familiar locations, participants frequently described the environment as entirely different, which triggered TTE. This phenomenon would suggest that the emergence of TTE is influenced more by individual interpretations of “difference” than by objective cultural disparity.

Speaking of the participants who achieved TTE through domestic travel, their descriptions often mentioned a deep appreciation for natural landscapes. In particular, TTE occurred in nature-rich locations such as Hokkaido and Okinawa. This result would suggest that visiting destinations with abundant natural resources may facilitate TTE for those traveling domestically.

Interestingly, the destinations where TTE did not occur were often the same as those where it did. Moreover, two of the four experience types identified from the descriptions of individuals who did not experience TTE overlapped with TTE-triggering factors, such as experiencing awe in nature and interacting with local people. This result suggests that individual differences play a significant role in whether TTE occurs, even when travelers encounter conditions that could trigger it. This finding aligns with previous research, which indicates that factors like personal traits and travel motivations can influence the likelihood of experiencing TTE (Zhao and Agyeiwaah, 2023; Nakajima and Takashi, 2023). However, the interaction between environmental and individual factors in the occurrence of TTE has yet to be fully clarified, emphasizing the need for more detailed investigation in future research.

Locations such as Thailand, Spain, Hiroshima, Tohoku, and Fukushima were major destinations where TTE occurred, but not for memorable travels without TTE. These results suggest that these locations were particularly conducive to TTE. In the case of Thailand, travelers who experienced TTE frequently noted differences in values between the local people and Japanese culture, as well as visible socioeconomic disparities and proximity to crime—elements that are less commonly encountered in Japan. From a global perspective, many countries would have cultures and environments that differ from Japan even more than Thailand. However, among the countries that are relatively accessible to Japanese travelers, Thailand may provide an optimal setting for experiencing a different culture and environment. Regarding Spain, participants who experienced TTE there mentioned being surprised by the locals’ relaxed and optimistic attitude. Given that Japanese society tends to emphasize diligence and seriousness (Takagi, 2022), encounters with such optimistic individuals as the Spanish (Schwarzer et al., 1997) may feel particularly novel, potentially making Spain a more conducive environment for transformative travel experiences.

Domestically, historical and disaster-related sites such as Hiroshima, Tohoku, and Fukushima were suggested to be conducive to TTE. This finding aligns with previous research indicating that dark tourism destinations, like Chernobyl, can foster transformative experiences (Zhuo et al., 2024). Visiting such sites may evoke strong emotional responses, facilitating deep reflection and personal transformation.

### 6.5 Transformation scale

Unlike the original six-factor structure, the TS for Japanese participants revealed a five-factor structure. Specifically, during the factor analysis, all three items related to spirituality (Q16, Q17, and Q18) and one introspection item (Q15) were removed, resulting in four excluded items. The remaining 14 items retain the same factor structure as the original version.

The lower mean scores for spirituality-related items compared to the Western individuals (Godovykh and Tasci, 2022) suggest that Japanese participants may have responded differently to these items. At first glance, the removal of these items seems to reflect a generally lower level of interest in religious or spiritual beliefs among Japanese people (Kobayashi, 2019; Pew Research Center, 2015). However, considering the present finding discussed in section 6.3, this may not necessarily imply that Japanese individuals do not experience spiritual changes following TTE; rather, these changes may not be identified as ‘spiritual’ within the Japanese cultural context. Therefore, to better capture such changes, it would be important to develop new items that can more effectively measure spirituality among Japanese participants, ensuring that terms like ‘religion’ and ‘spiritual’ are avoided, as these can feel unfamiliar or detached to many Japanese individuals.

The deletion of Q15, an introspection item (“I enjoy my privacy more than before”), may be explained by cultural differences in the concept of “privacy.” In Western contexts, privacy generally refers to time alone or with close family members (Solove, 2002), whereas in Japan, it often includes a variety of non-work-related activities, such as participation in hobby groups or community interactions (Morita, 1993). In this sense, privacy in Japan may involve social interactions rather than solitary introspection, depending on the context (e.g., hobbies). This cultural difference may have contributed to the inconsistencies within the introspection factor, leading to the removal of Q15.

Regarding the usefulness of TS in assessing the impact of TTE on Japanese participants, the current findings confirm the reliability and validity of the five-factor structure, indicating that TS is generally applicable to Japanese travelers. However, measuring spiritual changes with current TS items remains challenging. Future research should consider adding items with culturally appropriate wording to capture these aspects more effectively.

## 7 Limitations

This study has some limitations. First, since this study was cross-sectional, recall bias may have affected the responses. Future research should collect data longitudinally to examine the processes of TTE in greater detail with more reliable data. Second, the open-ended question regarding TTE outcomes may have emphasized psychological aspects, potentially discouraging participants from discussing changes in behavior, social dynamics, or spirituality. Future studies should address each of these aspects separately to capture a more comprehensive range of TTE outcomes. Third, while the findings suggest that TTE may lead to spiritual changes among Japanese participants, using terms such as “religion” or “spiritual” may limit effective responses, as many Japanese people may have reservations about these terms. Given the lack of guidance on how to phrase these questions to elicit open responses, future research could further explore this issue through unstructured interviews, which would allow for more flexible and exploratory communication.

## 8 Conclusion

Despite its limitations, this study provides evidence that travelers’ cultural backgrounds influence the nature of TTE. Specifically, the findings suggest that Japanese travelers tend to experience TTEs more frequently during business-related trips and that increases in humility resulting from TTEs are often accompanied by feelings of helplessness—patterns that differ from those observed among Western travelers. Future research should explore the detailed processes through which TTE is achieved and investigate the impact of cultural factors using a longitudinal study design. In addition, examining cultural influences across a broader range of countries is an important direction for future research.

## Data Availability

The raw data supporting the conclusions of this article will be made available by the authors, without undue reservation.
