# Supplementary material for: Japanese perspectives on transformative travel experience: humility, limitations, and dark tourism
Source: Front Psychol. 2025 Jun 23;16:1592027. doi: 10.3389/fpsyg.2025.1592027 (PMC12230070; doi:10.3389/fpsyg.2025.1592027)
Supplement: Supplementary file 1 [file Table_1.docx]

Supplementary Table 1

Informations regarding implementation of the present survey: following the checklist of Eysenbach (2004)

Item Category	Checklist Item	Explanations for the present survey
Design		
	Survey design	A cross-sectional survey. The sample was a convenience sample.
IRB		
	IRB approval	The present study was approved by the Research Ethics Committee, College of Contemporary Psychology, Rikkyo University (22-26).
	Informed consent	All participants provided informed consent through an opt-out process.As part of the opt-out procedure, participants received a document that included essential information to assist them in deciding whether to take part in the survey. This document outlined the survey's purpose, the estimated time commitment for participation, the voluntary nature of involvement, and the ability to withdraw consent at any time. Additionally, it was clarified that the results would be shared at conferences and published in academic papers, but no personal information that could identify individual participants would be revealed. It was also stated that the raw data would be kept for five years before being disposed of.
	Data protection	Participants' personal information was stored by the research company for compensation purposes. The data shared with the author did not include any identifiable personal information.
Developmemt and pre-testing		
	Development and testing	The electronic questionnaire was created by the research company, and the author collaborated in testing its usability and technical functionality.
Recruitment preocess and description of the sample having access to the questionnaire	Open survey versus closed survey	The present study was closed survey.
	Contact made	Initial contact with the participants was established online.
	Advertising the survey	The survey was announced as "Study on Travel and Values."
Survey administration		
	Web/Email	The company sent an email invitation for the current survey to its research monitors, and those who wished to participate clicked the survey link to fill out the online questionnaires.
	Context	The research company recruits survey monitors via its website, allowing interested individuals to register online. After registration, they will receive email notifications whenever surveys that meet their eligibility criteria are available.
	Mandatory/voluntary	Participation was voluntary.

Supplemenrtary Table 2
Demographic characteristics of the present participants.

		TTE-experienced		TTE-nonexperienced	
n		482		382	
Mean age (*SD*)		57.75 (13.83)		55.23 (13.29)	
Annual household income					
	Less than 1 million JPY	4.56	%	5.76	%
	1 million JPY to 2 million JPY	9.34	%	6.81	%
	2 million JPY to 3 million JPY	10.17	%	12.57	%
	3 million JPY to 4 million JPY	14.52	%	19.11	%
	4 million JPY to 5 million JPY	12.24	%	14.92	%
	5 million JPY to 6 million JPY	8.51	%	8.38	%
	6 million JPY to 7 million JPY	7.88	%	6.28	%
	7 million JPY to 8 million JPY	8.09	%	4.97	%
	8 million JPY to 9 million JPY	4.98	%	4.71	%
	9 million JPY to 10 million JPY	6.22	%	5.76	%
	10 million JPY to 15 million JPY	9.54	%	6.54	%
	15 million JPY to 20 million JPY	2.90	%	1.83	%
	20 million JPY to 25 million JPY	0.62	%	1.05	%
	25 million JPY to 30 million JPY	0.21	%	0.79	%
	More than 30 million JPY	0.21	%	0.52	%
Academic history					
	Junior high school	0.41	%	2.09	%
	High school	24.07	%	30.89	%
	Technical college/Junior college/vocational school	22.41	%	19.63	%
	Undergraduate	48.55	%	41.88	%
	Master's degree	3.11	%	3.66	%
	Doctor's degree	1.45	%	1.83	%
Number of travels in 2019 (*SD*)					
	Domestic	2.49 (4.21)		1.36 (2.47)	
	International	0.36 (0.88)		0.13 (0.63)	

Note. JPY is the abbreviation for "Japanese Yen".

Supplementary Table 3.
The top 15 most frequent extracted words (original Japanese words).

	Triggers of TTE	Frequency		Impacts of TTE	Frequency		Impressive experiences in the most memorable travel	Frequency
1	people (人)	59	1	world (世界)	76	1	sea (海)	24
2	see (見る)	50	2	think (思う)	58	2	for the first time (初めて)	22
3	Japan (日本)	42	3	self (自分)	56	3	see (見る)	18
4	culture (文化)	36	4	Japan (日本)	53	4	travel (旅行)	17
5	travel (旅行)	35	5	know (知る)	37	5	sight (景色)	16
6	for the first time (初めて)	34	6	feel (感じる)	36	5	go (行く)	16
7	international (海外)	33	7	people (人)	36	7	people (人)	15
7	naure (自然)	33	8	see (見る)	30	8	meal (食事)	13
9	sight (景色)	30	9	broad (広い)	29	9	beautiful (きれい)	12
10	world (世界)	27	10	country (国)	22	9	ride (乗る)	12
11	feel (感じる)	25	11	perspective (視野)	20	11	hot spring (温泉)	11
11	life (生活)	25	12	value (価値)	19	11	friends (友人)	11
11	experience (触れる)	22	13	spred (広がる)	18	13	tourism (観光)	10
22	know (知る)	22	14	travel (旅行)	18	13	nature (自然)	10
15	local (現地)	21	15	live (生きる)	17	15	local (現地)	9
			15	improtant (大切)	17	15	especially (特に)	9
						15	delicious (美味しい)	9
